# Study of Treatment and Reproductive Outcomes Among Reproductive-Age Women With HIV Infection in the Southern United States: Protocol for a Longitudinal Cohort Study

**DOI:** 10.2196/30398

**Published:** 2021-12-20

**Authors:** Anandi N Sheth, Adaora A Adimora, Elizabeth Topper Golub, Seble G Kassaye, Aadia Rana, Daniel Westreich, Jennifer Webster Cyriaque, Carrigan Parish, Deborah Konkle-Parker, Deborah L Jones, Mirjam-Colette Kempf, Igho Ofotokun, Ruth M Kanthula, Jessica Donohue, Patricia Raccamarich, Tina Tisdale, Catalina Ramirez, Lari Warren-Jeanpiere, Phyllis C Tien, Maria L Alcaide

**Affiliations:** 1 Division of Infectious Diseases Department of Medicine Emory University School of Medicine Atlanta, GA United States; 2 Infectious Diseases Program Grady Health System Atlanta, GA United States; 3 Division of Infectious Diseases Department of Medicine University of North Carolina-Chapel Hill Chapel Hill, NC United States; 4 Department of Epidemiology University of North Carolina-Chapel Hill Chapel Hill, NC United States; 5 Department of Epidemiology Johns Hopkins Bloomberg School of Public Health Baltimore, MD United States; 6 Department of Medicine Georgetown University Washington, DC United States; 7 Division of Infectious Diseases Department of Medicine University of Alabama-Birmingham Heersink School of Medicine Birmingham, AL United States; 8 Division of Oral and Craniofacial Sciences Department of Microbiology and Immunology University of North Carolina - Chapel Hill Chapel Hill, NC United States; 9 Department of Sociomedical Sciences Mailman School of Public Health Columbia University, NY United States; 10 Schools of Nursing, Medicine, and Population Health Sciences University of Mississippi Medical Center Jackson, MS United States; 11 Department of Psychiatry and Behavioral Sciences University of Miami Miller School of Medicine Miami, FL United States; 12 Departments of Epidemiology and Health Behavior University of Alabama-Birmingham Ryals School of Public Health Birmingham, AL United States; 13 Department of Nursing Family, Community & Health Systems University of Alabama-Birmingham School of Nursing Birmingham, AL United States; 14 Department of Pediatrics Georgetown University Washington, DC United States; 15 Division of Infectious Diseases Department of Medicine University of Miami Miller School of Medicine Miami, FL United States; 16 Department of Medicine University of California San Francisco San Francisco, CA United States; 17 Department of Veteran Affairs Medical Center San Francisco, CA United States; 18 Department of Obstetrics and Gynecology University of Miami Miller School of Medicine Miami, FL United States

**Keywords:** HIV, women’s health, depression, oral health, longitudinal cohort study

## Abstract

**Background:**

Nearly a quarter of the 1.1 million individuals with HIV in the United States are women. Racial and ethnic minority women in the Southern United States are disproportionately impacted. Reproductive-age women with HIV are prone to poor HIV outcomes but remain underrepresented in HIV research. We will answer contemporary questions related to the health outcomes in this population by enrolling a prospective cohort of reproductive-age women with and without HIV in the Southern United States.

**Objective:**

The Study of Treatment and Reproductive Outcomes (STAR) will enroll and retain 2000 reproductive-age women with and without HIV. The STAR will leverage the infrastructure of the US-based Multicenter AIDS Cohort Study (MACS)/Women’s Interagency HIV Study (WIHS) Combined Cohort Study, comprising the WIHS (a cohort of women with and at risk for HIV, which began in 1993), and the MACS (a cohort of gay and bisexual men with and at risk for HIV, which began in 1984). Although the advancing age of the participants enrolled in the MACS/WIHS Combined Cohort Study provides an opportunity to address the questions related to HIV and aging, the research questions pertinent to the reproductive years must also be addressed. The STAR will conduct high-priority scientific research in key areas with the overall aim of addressing the unique needs of reproductive-age women with HIV.

**Methods:**

The STAR is a prospective, observational cohort study that will be conducted at 6 sites in the United States—Atlanta, Georgia; Birmingham, Alabama; Jackson, Mississippi; Chapel Hill, North Carolina; Miami, Florida; and Washington, District of Columbia. Visits will occur semiannually for 2 years, with additional visits for up to 5 years. At each visit, the participating women will complete a structured interview for collecting key demographic, psychosocial, and clinical variables, and undergo biospecimen collection for laboratory testing and repositing (blood, urine, hair, vaginal, anal, and oral specimens). Pregnant women and infants will undergo additional study assessments. The initial scientific focus of the STAR is to understand the roles of key social determinants of health, depression, reproductive health, and oral health on HIV and pregnancy outcomes across the reproductive life span.

**Results:**

Enrollment in the STAR commenced in February 2021 and is ongoing.

**Conclusions:**

Through in-depth, longitudinal data and biospecimen collection, the newly initiated STAR cohort will create a platform to answer scientific questions regarding reproductive-age women with and without HIV. STAR will be uniquely positioned to enable investigators to conduct high-impact research relevant to this population. Building on the legacy of the MACS and WIHS cohorts, the STAR is designed to foster multidisciplinary collaborations to galvanize scientific discoveries to improve the health of reproductive-age women with HIV and ameliorate the effects of the HIV epidemic in this population in the United States.

## Introduction

### Background

Women of reproductive age are uniquely affected by the HIV epidemic. In the United States, nearly a quarter of the 1.1 million people living with HIV are women and most are racial or ethnic minority women in the Southern United States [1]. Reproductive-age women with HIV are highly vulnerable to poor health outcomes [2,3] and side effects of antiretroviral therapy (ART) [4,5], likely because of the structural, psychosocial, and biological factors, which manifest differently over time and across women’s reproductive experiences. Young racial or ethnic minority women with HIV are more likely to report lower retention in care and ART adherence, worse long-term clinical outcomes [6], and higher mortality [7] compared with men, White women, and older women. Outcomes vary across women’s reproductive lives; for example, although high levels of health care engagement and motivation for treatment adherence are documented during pregnancy [8], loss to follow-up [3,9], ART discontinuation [10], and lack of viral suppression [11] frequently occur during the postpartum period. These issues contribute to morbidity [12], mortality [13], ongoing HIV transmission risk to sexual partners [14] and future children, and lead to persistent disparities in perinatal HIV transmission [15]. Furthermore, women with HIV commonly face unplanned pregnancies [16,17] and adverse pregnancy outcomes [18].

Despite worse outcomes and unique challenges across multiple domains, the reproductive-age women with HIV in the United States remain underrepresented in HIV research [19,20]. Gaining a comprehensive understanding of the effects of HIV infection, reproductive health, and other key conditions in women of reproductive age is critical for developing future strategies to curb the HIV epidemic across populations in alignment with the US HIV National Strategic Plan [21].

The Southern United States is now the epicenter of the nation’s HIV epidemic. The 16 southern states and Washington, District of Columbia account for only 37% of the US population but 44% of all the people with HIV, and most new HIV diagnoses in the United States occur in the south: 8 of the 10 states and the 10 metropolitan statistical areas with the highest HIV rates are in the south [22]. Furthermore, 11 of the 13 states with the highest lifetime risk of HIV diagnosis are in the south [23], highlighting the urgent need to scale up the efforts to reduce the burden of the epidemic in this region. Geographic disparities observed in the HIV epidemic are particularly notable among women. Counties with the highest female-to-male HIV prevalence ratio are concentrated in the south; these counties have a higher proportion of people living in poverty and with lower education [24]. Of most concern, people with HIV in the south have very limited access to care [25], initiate ART at later stages [6], and have worse survival outcomes than people with HIV in other US regions [26]

The Women’s Interagency HIV Study (WIHS) [27], established by the National Institutes of Health (NIH) in 1993, was the largest and the longest running comprehensive prospective cohort study designed to investigate the effects of HIV infection on US women. The cohort included nearly 5000 women who completed biannual study visits with detailed structured interviews, physical, oral, and gynecologic examinations, laboratory testing, and specimen biobanking, and was managed by a robust data center. In 2019, the WIHS combined with the Multicenter AIDS Cohort Study (MACS) [28], a 30-year study of the effects of HIV infection on gay and bisexual men that enrolled over 7000 men with and without HIV, to form the MACS/WIHS Combined Cohort Study (MWCCS). The merger of MACS and WIHS sets a strong precedent for data harmonization and demonstrates the feasibility of pooling data from a cohort of reproductive-age women and the potential for important comparisons based on age and sex. Currently, women in the MWCCS are older (the median age of female participants is now 50 years) and do not adequately represent the contemporary population of women of reproductive age. The Study of Treatment and Reproductive Outcomes (STAR) will fill this gap by establishing a new cohort of reproductive-age women with and without HIV, a group that is underrepresented in HIV research, to address issues that uniquely affect this population, and to assess outcomes across women’s reproductive life span. In addition to the establishment of the cohort, the STAR will address health conditions that are highly prevalent, understudied, and likely to be linked to poor health and HIV outcomes among women with HIV—mental health and oral health—as its initial scientific focus.

**Figure 1:**
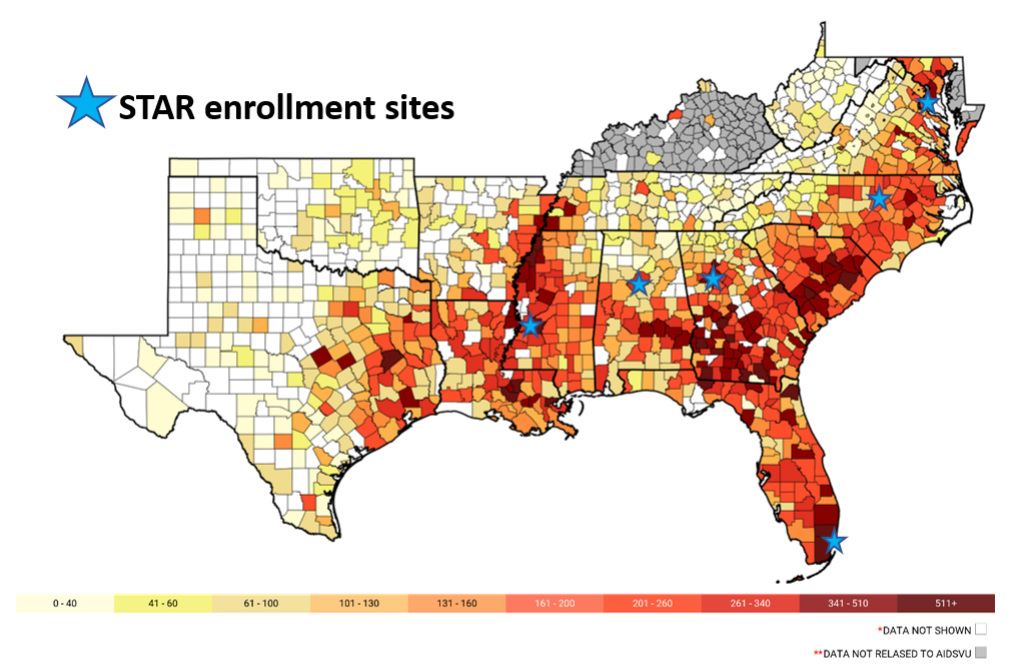
Southern United States with county-level rates of women with HIV per 100,000 persons in 2018. Source: AIDSVu, Emory University, Rollins School of Public Health [29]. *Data not shown to protect privacy because of a small number of cases and/or a small population. **State health department, per its HIV data rerelease agreement with CDC, requested not to release data to AIDSVu. There are no county-level maps for Alaska, District of Columbia, and Puerto Rico because there are no counties in these states. STAR: Study of Treatment and Reproductive Outcomes.

The 6 STAR sites, located in the Southeastern United States, provide optimal settings for the recruitment and retention of young women with and without HIV into a new longitudinal cohort study focused on reproductive-age women. The STAR sites are located in Atlanta, Georgia; Birmingham, Alabama; Jackson, Mississippi; Chapel Hill, North Carolina; Miami, Florida; and Washington, District of Columbia (Figure 1 [29]). These sites include 3 of the 5 metropolitan areas with the highest HIV rates in the United States (Miami [first], Atlanta [third], Jackson [fourth]) [1], and all have been recently prioritized in the Health and Human Services’ *Ending the HIV Epidemic* initiative [29]. The STAR will recruit and follow 2000 reproductive-age women with and at risk for HIV infection to address key scientific questions in this understudied population. The STAR will open a new line of scientific investigation to address the unique health challenges of women spanning the reproductive life span. Using approaches similar to those used by the MWCCS (including its standardized assessments, clinical sites, data management approach, and strong community partnerships), the STAR will rapidly achieve its recruitment targets. In this study, we describe the research protocol for establishing this new longitudinal cohort.

### Key Scientific Objectives of the STAR

The overall objectives of this study are (1) to create a platform for multi- and cross-disciplinary research by enrolling a representative cohort of women with and at risk for HIV and collecting longitudinal clinical, behavioral, and laboratory data to investigate key issues across women’s reproductive lives using novel scientific approaches; (2) to understand the impact of depression on critical HIV-related, pregnancy-related, and other reproductive health outcomes across the reproductive life span; and (3) to investigate the role of key social determinants of health, HIV-related factors, and pregnancy on oral health among women. The long-term goal is to use results from the STAR to develop strategies to improve the health of women with HIV, their children, and their communities.

Depression is highly prevalent among women with HIV and the prevalence of depressive symptoms among these women is higher than that in the general population [30]. Co-occurrence of HIV infection and depression contributes to poor health outcomes, including suboptimal ART adherence [31], substance use [31-33], sexual risk behaviors [34], accelerated HIV disease progression, viral nonsuppression, HIV-related and all-cause mortality [35-39], and adverse maternal and child health outcomes [40-44]. Reproductive-age women with HIV are at higher risk for depression-related adverse outcomes and are less likely to seek mental health services than older women, owing to stigma, lack of awareness, limited resources, and competing childcare and other obligations [34,45,46]. Depression is of particular concern for pregnant women with HIV; perinatal depressive symptoms have been reported in nearly half of the women with HIV compared with 10%-20% in the general population [40]. We seek to understand the effects of depression on HIV and reproductive health outcomes, and how these relationships are modified by individual and socio-contextual factors and their interacting combinations. We hypothesize that depression, especially during and after pregnancy, is associated with poor HIV and reproductive health outcomes and its effects are modified by individual- and community-level factors.

Racial and ethnic minority women experience a higher burden of oral disease compared with their White counterparts, and these health disparities persist and are augmented in the context of HIV infection [47-49]. Common oral health conditions are gingival inflammation and periodontal disease, which are more frequent and severe among persons with HIV [49,50] and may influence pregnancy outcomes [51-56]. Previous research on women with HIV have demonstrated the associations between oral health and other nonoral health outcomes, low dental services access and use [57-59], and have shown that high rates of periodontal disease are associated with systemic inflammation among persons with HIV [49]. In this study, we will evaluate the intersecting relationships of depressive symptoms, reproductive health, oral health, and pregnancy outcomes and their mediators across the reproductive life span of women with HIV. We hypothesize that oral health indicators (such as periodontal disease and oral health-related quality of life [OHRQOL]) are influenced by HIV-related factors and social determinants of health, particularly during and after pregnancy.

## Methods

### Study Overview and Design

The STAR is a longitudinal observational study that will include 2000 women aged 18-45 years at the time of enrollment who have been diagnosed with HIV infection or are HIV-seronegative but at risk for HIV infection. To evaluate the effect of HIV status on key study variables, 1500 participants with HIV and 500 participants without HIV who meet the inclusion criteria will be enrolled.

**Figure 2:**
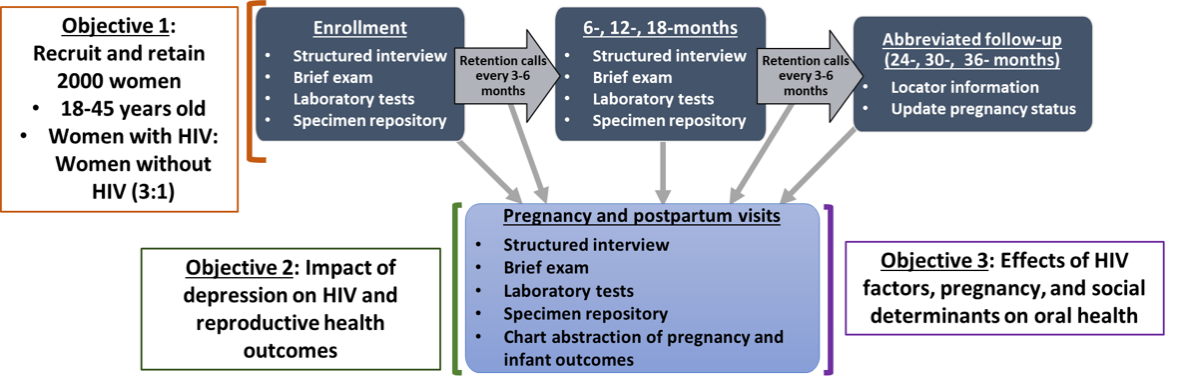
Overview of the Study of Treatment and Reproductive Outcomes objectives and study visits.

Comprehensive study visits will occur at the time of enrollment and at 6, 12, and 18 months thereafter. Semiannual brief telephone or web-based visits will continue until the end of follow-up (ie, up to 5 years) to facilitate retention and identify women who become pregnant. Women who become pregnant during follow-up will have additional pregnancy and postpartum study visits that comprise of interviewer-administered questionnaires, biological specimen collection, and medical record abstraction (Figure 2). Selected mental and oral health measures and biospecimens (including oral specimens) will be included to assess the key scientific objectives of investigating depression and oral health. Additional data and biospecimens will be available for future studies aimed at addressing key research priorities for women with HIV.

### Consent and Institutional Review Board Approval

The study was reviewed and approved by the STAR single Institutional Review Board (sIRB) at the University of Miami (sIRB# 20190953) that provides regulatory oversight over all the participant recruitment sites. Informed consent will be obtained from all willing participants before enrollment. Any substudies that require additional questionnaires or measures will be submitted as an amendment or as a separate study protocol and additional consent will be obtained as determined by the sIRB. Consent forms and questionnaires will be administered in English or Spanish, according to each participant’s preference.

### Participants

All the participants must meet the following eligibility criteria: (1) aged between 18 and 45 years, (2) be female at birth, (3) able and willing to give informed consent, (4) able to complete the interview in English or Spanish, (5) able and willing to have blood drawn and stored, and (6) agree to medical record abstraction to ascertain pregnancy-related outcomes (including infant demographics and clinical characteristics). Participants with HIV must have evidence of HIV infection, as documented by any Food and Drug Administration–approved assay.

Participants without HIV must report at least one of the following high-risk exposures within 5 years before enrollment: (1) sex with ≥3 men; (2) use of crack, cocaine, opiates, methamphetamines, or any injection drug use; (3) sexually transmitted infection (STI) diagnosis; (4) sex for drugs, money, or shelter; (5) sex with a man with known HIV infection; or (6) a male sexual partner who reports one of the following within the last 5 years before enrollment: (a) use of crack, cocaine, opiates, methamphetamine, or injection drugs; (b) STI diagnosis; (c) sex with ≥3 people; (d) sex for drugs, money, or shelter; (e) sex with an individual with known HIV infection; or (f) any prior incarceration. Similar inclusion criteria were previously used to enroll HIV-seronegative women for WIHS and resulted in the enrollment of a group of HIV-seronegative women who were well-matched to women with HIV with respect to demographic, behavioral, and other risk characteristics [27]. Participants will be excluded if they are planning to move out of the area during the study follow-up period, if the study procedures are not completed at any of the study sites, or if they have any condition that would make participation in the study unsafe or interfere with achieving the study objectives.

### Recruitment

The recruitment of eligible study participants will be distributed across the 6 study sites and is expected to occur over the next 3 years. The STAR sites and the data center will review and monitor the enrollment demographics. The recruitment strategy is designed to ensure recruitment of women who are (1) representative of the local epidemic with regard to race or ethnicity and other sociodemographic factors, (2) committed to participate in all the study-related activities, and (3) willing to commit to long-term follow-up. Participants will be recruited from (1) clinical sites (adult or women’s, obstetric, adolescent, and pediatric HIV clinics), (2) current MWCCS participants, (3) other existing local research studies, and (4) community locations (ie, community-based organizations and clinics). All the participating study sites have forged deep relationships with clinical sites and community advocacy groups to facilitate clinic- and community-based recruitment of diverse populations of women with and without HIV. We will iteratively review our procedures in collaboration with our community partners during the enrollment period to optimize and maintain effective recruitment strategies to meet the recruitment targets.

### Incentives

All the participants will be offered appropriate compensation for their time and effort. Compensation will vary according to the study procedures completed at the respective visit and may include reimbursement for travel to and from the location of the study clinic.

### Study Procedures

Screening and enrollment may be conducted in 1 visit. Data collection and procedures will include participants’ demographics and residential addresses for geocoding, a series of interviewer-administered questionnaires (including medical, reproductive health, sexual risk behavior, medication adherence, psychosocial variables, oral health, and mental health), limited physical examination (blood pressure, pulse, height, and weight), and biological specimen collection (including blood, urine, hair, and oral, anal, and vaginal specimens). Mucosal swabs (oral, anal, and vaginal) will be self-collected but may be clinician-collected (if self-collection is not feasible or based on the participant’s preference). Participants will be asked to attend semiannual study visits for at least 18 months and will be contacted every 3-6 months thereafter to facilitate study retention for the duration of the study period. Women may be asked to attend additional visits during pregnancy or for substudies, and medical record abstraction will be used to collect pregnancy, birth, and infant outcomes data. To evaluate the intersecting relationships between oral disease and other health outcomes in reproductive-age women with and without HIV, an additional dental plaque specimen will be collected from a subset of pregnant women as part of an oral substudy.

All the sites in this multicenter study use a common protocol, consisting of the same questionnaires; specimen collection, processing, storing, and shipping protocols that meet federal standards for all specimen types; and centralized data management, hosted at Johns Hopkins University. The study personnel will be trained to perform all assessments. All the specimen collection, handling, processing, and storage will be conducted according to written standard operating procedures. All the study laboratories will maintain the required certifications and adhere to best practices. The sites will have site-specific standard operating procedures for the notification of clinically relevant laboratory results and referral for appropriate clinical follow-up (if required). All the specimens will be tracked using the laboratory information management system to manage the collection of biological specimens. Tracking will be centralized using unique STAR study ID and specimen identification numbers assigned by the data center.

### Description of Study Assessments

Data collection will include a structured interview and limited physical assessments (Table 1). Specific to the key scientific objectives of the STAR, depressive symptoms will be measured using the widely used Center for Epidemiologic Studies Depression scale for all the participants and with the Edinburgh Postpartum Depression Scale administered to the participants who are pregnant or within 12 months of delivery, as continuous scores and categorized by key cutoffs [27,40,60,61]. We will also obtain supplemental mental health histories and the related medication use through questionnaires. Oral health histories will be collected using validated questionnaires previously used in WIHS [57-59] and OHRQOL information will be collected using the Oral Health Impact Profile [62-64]. Clinical outcomes will be abstracted from the medical records, and biological specimens described above will be collected for laboratory testing and repository storage (Table 1). The following biological specimens will be stored in the local biospecimen repository of each study site using standardized methods and will be shipped at 3-month intervals to an NIH-sponsored central repository for long-term storage: blood (plasma, serum, and peripheral blood mononuclear cells), saliva, urine, mucosal specimens (vaginal, oral, and anal), and hair. If the interview components or biospecimens cannot be completed or collected, respectively, at the designated visit, then a participant may return or be contacted to complete the visit elements within 90 days of the visit initiation.

**Table 1:** Study of Treatment and Reproductive Outcomes schedule of assessments.

Visit		Baseline	Baseline+6 months	Baseline+12 months	Baseline+18 months	Baseline+24, 30, 36 months
**Interview**						
	Sociodemographics	X^a^	X	X	X	P^b^
	Medical or health history, including HIV history and treatment, pregnancy history, and mental health history	X	X	X	X	P
	Obstetric, gynecologic, and reproductive health history (includes pregnancy intentions)	X	X	X	X	P
	Contraception access	X	X	X	X	P
	Use of Health care	X	X	X	X	P
	Oral health questionnaire^c^	X	—^d^	X	—	—
	Behavior form^e^	X	X	X	X	P
	Pregnancy or postpartum form^f^	P	P	P	P	P
	Drug Abuse Screening Test-10 [65,66]	—	X	*^g^	*	*
	Lifetime discrimination [67] and stigma [68]	—	X	*	*	*
	History of abuse [69]	—	X	*	*	*
	COVID-19 questionnaire	X	—	X	—	—
	Abbreviated interval health (medication changes, pregnancy, contraception)	—	M^h^	M	M	X
**Outcomes ascertainment**						
	Outcomes ascertainment form^i^	X	X	X	X	X
	Pregnancy ascertainment form	P	P	P	P	P
	Infant ascertainment form	P	P	P	P	P
**Physical examination**						
	Height, weight, blood pressure	X	* or P	X	* or P	—
	Brief oral exam with dental photograph review	X	P	P	P	P
**Laboratory assessments**						
	CBC^j^, differential	X	*	—	—	—
	CD4/CD8 panel (women with HIV only)	X	*	X	* or P	—
	HIV RNA (women with HIV only)	X	*	X	* or P	—
	HIV antibody or antigen test (HIV only)	X (if last test >3 weeks)	*	X	* or P	—
	Comprehensive metabolic panel	X	*	—	—	—
	HgA_1c_^k^ and lipids	X	*	—	—	—
	STI^l^ (chlamydia, gonorrhea, trichomonas)	X	*	X	—	—
	Syphilis test	X	*	X	—	—
	HPV^m^	X	*	X	—	—
	Urine pregnancy screen	X	* or P	X	* or P	—
**Biospecimens for repository**						
	Serum, plasma and cells (PBMC^n^)	X	* or P	X	* or P	P
	Urine supernatant	X	* or P	X	* or P	P
	Vaginal, oral, rectal self-swab	X	* or P	X	* or P	P
	Unstimulated saliva	X	* or P	X	* or P	P
	Hair (women with HIV only**)**	X	* or P	X	* or P	P
	Dental plaque	P	P	P	P	P

^a^X: Form completed at the visit.

^b^P: Additional forms to be completed if the participant meets criteria for a *pregnancy visit* or a *postpartum visit.*

^c^Includes Oral Health Impact Profile [62-64], dental neglect [70], and oral health-related quality of life [71-74].

^d^Assessment not performed at this visit.

^e^Includes the following topics: sexual behavior, alcohol use, substance use, tobacco use, Center for Epidemiologic Studies Depression [60], loneliness [75], social support [76], perceived stress [77], quality of life [78], attitudes toward aging [79], resilience [80], sense of community [81], safety [82], and perceptions on *undetectable=untransmittable.*

^f^Includes Edinburgh Postnatal Depression Scale [61] (pregnancy and postpartum) and intimate partner violence (postpartum)[69].

^g^If not completed at the preceding visit in which assessment was scheduled as noted by “X”.

^h^M: Additional forms to be completed if the participant is unable to come in for an in-person visit.

^i^Includes HIV history and treatment, cervical health (Papanicolaou test and human papillomavirus testing and vaccination, dysplasia, and cancer), sexually transmitted infection history, cancer, and death.

^j^CBC: Complete blood count.

^k^HgA_1c_: hemoglobin A_1c_.

^l^STI: sexually transmitted infection.

^m^HPV: Human papillomavirus.

^n^PBMC: Peripheral blood mononuclear cells.

For geocoding, the STAR staff at each site will collect the addresses from the participants who have consented to geocoding, geocode them (using ArcGIS; Esri), and assign a census block group. ArcGIS will match each participant’s geocoded location to a Federal Information Processing Standard (FIPS) code that identifies geographic locations in the United States. Each site will create a limited data set that contains only the participants’ FIPS codes and the study ID. This limited data set will be securely transferred to the University of North Carolina at Chapel Hill site, which will link the FIPS codes to census-linked data sets, such as the American Community Survey or Decennial Census, to create group-level variables that will describe the locations where participants live.

The participants with laboratory-confirmed pregnancies will be asked to undergo pregnancy-specific assessments either during a scheduled core visit (baseline, 6, 12, and 18 months) or at an additional visit scheduled after 20 weeks of gestation. All the pregnant participants who reach beyond 20 weeks of gestation will have a postpartum visit conducted approximately 3-6 months after delivery. For each participant who reports pregnancy that reaches beyond 20 weeks of gestation, the clinical and laboratory data related to the pregnancy will be obtained using standardized chart abstraction and will include the following obstetric outcomes: prenatal or postpartum visits attended, hypertensive disorders (including preeclampsia and eclampsia), gestational diabetes, miscarriage, preterm birth, stillbirth, and fetal growth restriction and the following infant outcomes: gestational age at birth, birth weight, length, head circumference, birth defects, newborn screen, infant prophylaxis regimen, anemia, lead levels, and Apgar scores.

### Adaptations During the COVID-19 Pandemic

In light of the COVID-19 pandemic, safety measures will be taken to protect the study participants. Owing to the heterogeneity of the effects of the pandemic within the United States, each site will follow local guidance on the resumption of in-person visits using safety measures to protect the participants and the staff. To facilitate research productivity, virtual visits through phone or an Institutional Review Board–approved remote communication and signature platform will be conducted when in-person study visits are deemed unfeasible. The sites will be approved to implement remote consent procedures to limit the in-person contact time. Options to obtain consent will include (1) remote consent process using secure platforms that can handle protected health information for communication or electronic documentation (ie, Zoom, RedCap, and DocuSign) with institutional approval or (2) verbal consent process in which the script of the verbal consent is read slowly and clearly to the participant by trained study staff. If the potential participant agrees to participate in the research interview, the person obtaining the consent will sign and date the verbal consent script. The study staff will reiterate that the participant will be asked to sign the full consent documents during the first in-person visit. Verbal consent will be obtained only for administering the questionnaires. After the remote portion of the study visit is completed, the participants will have an in-person visit to complete the physical examination elements including the collection of biological specimens. As part of the COVID-19 pandemic modifications to the protocol, the participants will be asked to self-collect unstimulated saliva in a private room. In addition, we will incorporate a brief COVID-19 questionnaire to capture data on the prevalence of COVID-19 and vaccination among the study participants.

### Data Management

A confidential research record with all the source documents and data will be maintained for each study participant. Each participant will be assigned a unique study ID number at the time of enrollment that will be used in all subsequent and associated study documents and biospecimens. Any data (eg, medical records) that are acquired with identifiers will be maintained at the site and stored securely with limited access to specified site study personnel. The data collected will be entered directly during the interview by the study staff into a centralized, web-based data management system called GEMINI, which is developed and maintained by Johns Hopkins University. Each STAR site will work with the centralized data center to create and maintain data quality. The data center will provide centralized data management training, including study procedures for data collection, entry, checking for completeness and skip patterns, common mistakes, and error correction. When direct data entry is not feasible, paper forms will be used and data will be subsequently entered. The data center personnel at Johns Hopkins University will not interact with the participants and will not receive identifiable data, specimens, or the ID code link of the participants. Once all the data from a visit are entered, the data for that visit will be frozen and will undergo a rigorous central editing and quality-checking procedure to ensure the highest quality of every file that is stored. The data center will apply central editing programs at the end of each visit. Once the sites respond to edit queries through GEMINI, the data from a visit will be merged with the master longitudinal database.

### Study Leadership and Investigator Procedures

The principal investigators of the STAR will lead the operations at their respective sites and contribute to the scientific research agenda based on their complementary expertise. The STAR Executive Committee (EC) will work collaboratively with the MWCCS EC, the data center, community stakeholders, and NIH representatives. The STAR EC will meet twice every month through teleconference and with the MWCCS EC during the semiannual meetings. The STAR investigators will engage the community stakeholders to provide input on study design, data interpretation, and dissemination of findings during the MWCCS semiannual meetings and as part of the local MWCCS and STAR community advisory board activities.

We have assembled a Scientific Advisory Group (SAG) of highly accomplished external investigators who are engaged in research with reproductive-age women with HIV to (1) create a collaborative framework for establishing a rigorous scientific agenda, (2) provide a venue for participation and access to the cohort to a wider group of investigators, and (3) foster systematic interaction with established investigators who complement the scientific expertise of the STAR investigators. The SAG members will participate in quarterly conference calls and an annual meeting.

In addition to the planned scientific objectives, the STAR will emphasize multidisciplinary collaborations in women’s health and HIV/AIDS research among leading scientific experts across all the sites to facilitate applications for research to define and understand health outcomes over the course of women’s reproductive live span. To achieve this goal, the STAR will leverage the scientific cores which already exist because of MWCCS and each STAR site will have a scientific core focused on promoting high-priority scientific research at the site led by its STAR principal investigator. The STAR SAG will also advise on establishing a research agenda that will be informed by the research priorities of the Office of AIDS Research. External investigators who wish to use the STAR data or specimens will submit a request in collaboration with the STAR investigators. Once the request is approved, the data center will coordinate the release of deidentified data and specimens.

### Data Analysis

#### Sample Size

Owing to the anticipated size of the cohort (larger than the sample size used in a previous analysis of depressive symptoms [83]) and the high prevalence of depression among young women with HIV (over 20%), we expect more than sufficient power for the primary analyses. We anticipate that approximately 10% of the participating women will become pregnant during follow-up [84], of whom more than 40% are likely to experience postpartum depression, which will provide >80% power to detect a risk ratio of 2 for common obstetric outcomes (overall incidence>15%). The power will increase if more pregnancies are observed.

#### Outcomes

HIV outcomes across several planned analyses will include ART adherence (by self-report), retention in care (2 HIV care visits 90 days apart within 1 year), and durable viral suppression (HIV-1 RNA<200 copies/mL at all visits). Reproductive health outcomes will include pregnancy intentions, contraception access and use, STI acquisition, and pregnancy outcomes ascertained from medical records including adherence to prenatal care, preeclampsia, eclampsia, gestational diabetes, miscarriage, preterm birth, and fetal growth restriction.

#### Statistical Analysis

We will use descriptive statistics to understand the distribution of the baseline variables, exposures, and outcomes. To understand the role of depression in this cohort population, regression models will be used to examine whether individual- and community-level characteristics are associated with the incidence and prevalence of depressive symptoms, accounting for clustering by neighborhood and when appropriate, using generalized estimating equations and robust variance estimators. We will estimate the effect of depression on the outcomes and estimate how this relationship may be mediated by other individual- and community-level factors. Causal diagrams will be informed by prior literature and the Modified Social Ecological Model [85], which highlights the multilevel risks and contexts of HIV infection and situates individual behavior within the social, structural, and policy contexts.

We will also estimate both the effects of exposures (eg, depression) and the effects of potential interventions on those exposures (eg, cognitive behavioral therapy) using both traditional regression approaches and 2 modern epidemiological analysis methods: inverse probability weighted marginal structural models [86-88] and the g-formula [89,90].

We will also use these data to investigate (1) the effects of social determinants (including dental care access or use, depression, and substance use, including opioids), psychosocial factors (depression, anxiety, medical mistrust, smoking, and substance use), and HIV factors (ART use, CD4+ T lymphocyte count, and HIV viral load) on 2 main oral health indicators—periodontal disease and OHRQOL; and (2) the associations between periodontal disease and pregnancy outcomes (preterm birth, hypertensive disorders of pregnancy, and low birth weight). We will use longitudinal mixed methods models to examine these associations with periodontal disease and OHRQOL. Among women who experience pregnancy, we will use logistic regression models, adjusting for covariates (including those associated with periodontal disease), to examine the association between periodontal disease and pregnancy outcomes.

### Integration and Dissemination of Findings

Findings from all the proposed analyses will be disseminated to the STAR investigators, SAG members, community partners, and NIH representatives. The findings will also reach a broad scientific audience through presentations at national and international conferences and publication in peer-reviewed journals.

## Results

Research activities for this study commenced in September 2019 and are ongoing. Participant enrollment and data collection commenced in February 2021 and are ongoing. As of July 15, 2021, 165 participants have been enrolled across the STAR sites (142/165, 86.1% non-Hispanic Black; 10/165, 6.1% non-Hispanic White; and 10/165, 6.1% Hispanic or Latina women, similar to the race and ethnicity distribution of participants previously recruited for WIHS at the same sites [27]). Data analysis is scheduled to begin next year. Owing to the scientific engagement efforts, additional scientific questions to understand genital and extragenital STI prevalence and incidence in this cohort were added as part of a substudy. Furthermore, a substudy has also been added to describe the use of the human papillomavirus vaccine and its association with human papillomavirus infection and type among the STAR participants. Scientific engagement efforts are ongoing to expand the research agenda of the STAR.

## Discussion

Despite the availability of ART, reproductive-age women with HIV experience suboptimal viral suppression and engagement in HIV care, especially among racial or ethnic minority women in the Southern United States [91]. This cohort will be uniquely positioned to enable investigators to conduct high-impact research relevant to reproductive-age women with HIV, including research related to pregnancy. By leveraging strategies from the MWCCS, this cohort will be rapidly established despite the challenges posed by the COVID-19 pandemic and will obtain high-quality data using established and validated surveys, specimen collection procedures, and data management tools. When relevant, assessments will also be harmonized with those used in other US cohorts of pregnant and reproductive-age women with HIV to allow for potential future collaborations and data synergy. Adaptation of study procedures will allow remote visits and specimen collection while following pandemic procedures at the sites, enabling timely enrollment and progress. The potential impact of this cohort is evidenced by the additional supplements awarded before the start of participant enrollment to address additional scientific priorities for this population. This study will produce high-impact results with the potential to improve the health of reproductive-age women with and without HIV in the Southern United States, optimize the gains of ART, and ameliorate the effects of the epidemic in this population.

## Abbreviations

ART: antiretroviral therapy
EC: Executive Committee
FIPS: Federal Information Processing Standard
MACS: Multicenter AIDS Cohort Study
MWCCS: Multicenter AIDS Cohort Study/Women’s Interagency HIV Study Combined Cohort Study
NIH: National Institutes of Health
OHRQOL: oral health-related quality of life
SAG: Scientific Advisory Group
sIRB: Single Institutional Review Board
STAR: Study of Treatment and Reproductive Outcomes
STI: sexually transmitted infection
WIHS: Women’s Interagency HIV Study
